# Effect of Jian-Pi-Zhi-Dong Decoction on the Amino Acid Neurotransmitters in a Rat Model of Tourette Syndrome and Comorbid Anxiety Disorder

**DOI:** 10.3389/fpsyt.2020.00515

**Published:** 2020-06-05

**Authors:** Wen Zhang, Wenjing Yu, Xiaofang Liu, Qian Wang, Xue Bai, Xia Cui, Sumei Wang

**Affiliations:** ^1^Department of Pediatrics, The Third Affiliated Hospital, Beijing University of Chinese Medicine, Beijing, China; ^2^Department of Pediatrics, Dongfang Hospital, Beijing University of Chinese Medicine, Beijing, China

**Keywords:** Tourette syndrome, comorbidity, Jian-Pi-Zhi-Dong Decoction, Traditional Chinese Medicine, glutamate, γ-aminobutyric acid, N-methyl-D-aspartate receptor, GABA receptors Type A

## Abstract

Amino acid neurotransmitters have been shown to correlate with Tourette syndrome (TS) and its comorbidities. In this study, we investigated the effects of Jian-Pi-Zhi-Dong Decoction (JPZDD), a formula containing 10 different Chinese medical herbs, on amino acid neurotransmitters in rats. We established a rat model of Tourette syndrome and comorbid anxiety with an iminodipropionitrile injection plus uncertain empty water bottle stimulation for 3 weeks. Then the rats were randomly divided into four groups: control group and model group were gavaged with saline, while the remaining two treatment groups were gavaged with fluoxetine hydrochloride or JPZDD for four consecutive weeks. We recorded the behaviors of the rats with TS and comorbid anxiety by stereotypy recording, open field test, and elevated plus maze. We observed mitochondrial changes with transmission electron microscopy. We measured the content of glutamate (GLU) and γ-aminobutyric acid (GABA) both in the serum and striatum and the expression of their receptors by Western blot and real-time polymerase chain reaction. The study revealed that JPZDD was effective in alleviating the behavioral symptoms of both tic and anxiety in the rat model groups. These results might be associated with the increase in GABA levels and decrease in GLU levels in the serum, as well as an increase in striatal GABA level by the activation of GABA receptors Type A (GABAAR). JPZDD treatment also reversed the mitochondrial dysfunction both in the striatum and cortex in affected animals.

## Introduction

Tourette syndrome (TS) is a neuropsychiatric condition with the manifestation of multiple, spontaneous movements and vocalizations called tics. Normally, TS is diagnosed by the onset of multiple motor tics and vocal tics lasting at least 1 year in clinic. The different tics do not need to present concurrently, but manifest at some point throughout the course of the illness (1). The disorder is usually self-limiting, but may continue into adulthood. TS is also characterized by high rates of psychiatric comorbidities. The prevalence of comorbidities among TS patients is 85.7% (2). Among the comorbidities, around two thirds of patients had attention deficit hyperactivity disorder (ADHD) or obsessive compulsive disorder (OCD), and around one-third of patients had different kinds of mood disorders, such as anxiety, and disruptive behavior (3). The clinical impact of co-occurring conditions may be greater and compromise an extra burden much more than the tics (2). The comorbidities represent greater impairment. Treatment of comorbidities is crucial for all patients with TS, but few studies have fully characterized these comorbidities. The purpose of this study was to determine if Chinese traditional medicine could affect neurotransmitters and mitochondrial function in model rats with TS and comorbid anxiety.

The prevalence of anxiety in TS patients changes greatly depending on the different age level and methodologies used (4). For example, the ratio of TS comorbid generalized anxiety disorder has ranged from 19% to 80%, with high incidence rates in children and youth (5). There is a positive correlation between the degree of anxiety and the degree of the tic; a higher degree of anxiety was associated with more severe tic symptoms (6). Cortico-striato-thalamo-cortical (CSTC) circuits link specific regions in the frontal cortex to subcortical structures (including the basal ganglia). Lower white matter volume in prefrontal cortex can be seen in TS (7). Evidence supports that the involvement of CSTC circuits could provide the feasibility for understanding TS (8). Several neurotransmitters, including dopamine, Glutamate (GLU), and γ-aminobutyric acid (GABA) are active within CSTC circuit and neurotransmitter abnormality has been considered to be relevant in the pathophysiology of tics (9). For example, abnormalities of GABA and GLU have been found to be closely associated with TS (9). When GLU or GABA is released into the synaptic cleft, it binds its receptors in the postsynaptic membrane to exert biological effects. GLU, which is the primary excitatory neurotransmitter, is an essential component of the CSTC pathway implicated in TS. In postmortem examination of TS patients, reduced GLU levels in the brain could evidence the involvement of GLU system dysfunction within CSTC pathways (10). The N-methyl-D-aspartate receptor (NMDAR) is a main but non-exclusive receptor of GLU. Targeting selective subunits of the NMDAR or release-modulating GLU autoreceptors provides a new method to modulate the dysfunction of GLU neurotransmission in TS patients (11). One proposed hypotheses in this study is that the enhancement of neurotransmission at the NMDAR would be beneficial in TS (12). GABA is the primary inhibitory neurotransmitter located in both the striatum and cortex. Once the GLU/GABA balance within the striatum is disrupted, tic-like behavior can cause. So GABA signaling is thought to play a key role in the inhibition control of TS (13). GABA regulates brain excitability *via* its GABA_A_ receptors. Postmortem examination of a brain with TS has demonstrated that altered GABA receptors Type A (GABAAR) binding within the striatum is involved in the pathogenesis of TS (14). In fact, not only GABA and GLU neurotransmitter but also their various receptors are supposed to potential therapeutic targets of TS and its comorbidities. Major psychiatric illnesses have traditionally been viewed as “neurochemical diseases,” these disorders are associated with mitochondrial disorders. The mitochondria are essential for not only energy metabolism but also neurotransmission. The function of mitochondria influences neurotransmission mainly by promoting short- and long-term neuronal plasticity, adjusting cellular resilience to stress and behavioral adaptation (15). New research reports that TS also involves a mitochondrial disorder (16).

Traditional Chinese medicine (TCM) is applied to the treatment of TS and its comorbidities in Chinese clinics. The Jian-Pi-Zhi-Dong Decoction (JPZDD) is derived from a modification of Liu-Jun-Zi-Tang (LJZT) and Xie-Qing-Wan (XQW), which contains 10 ingredients. JPZDD has displayed not only anti-tic properties, but also properties that help treat mood disorders in clinic (17). Although early reports have demonstrated JPZDD could modulate the balance of excitatory and inhibitory neurotransmission in TS rats (18), we have been unable to find any study that investigates the potential effect of JPZDD on mitochondrial function and the neurotransmitters in a rat model with TS and comorbid anxiety. In the present work, our model used 3,3-iminodipropionitrile (IDPN) injection combined with uncertain empty bottle stimulation (19), aiming to verify that the beneficial effects of JPZDD on both tics and anxiety mainly through alleviating brain mitochondrial dysfunction and keeping the balance of neurotransmitters by its receptors.

## Materials and Methods

### Experimental Animals

Male Sprague Dawley rats (n = 48, 3 weeks old, 50 ± 10 g) were purchased from Beijing Vital River Laboratory Animal Technology Co., Ltd. (Beijing, People's Republic of China; No SCXK 2012-0001). All the experimental animal procedures conformed to the guidelines of the Beijing University of Chinese Medicine Animal Care and Use Committee. All experimental protocols were reviewed and approved by the Animal Experimentation Ethics Committee at the Beijing University of Chinese Medicine (No: BUCM-4-2019042503-2098). All the work tried to minimize suffering. The animals were maintained at 21°C ± 1°C in a standard 12-h light/dark cycle with their environment maintained at the relative humidity of 30% to 40%.

### TS and Comorbid Anxiety Model

The rats were provided food and water freely 7 days before the beginning of the experiment. The rats were randomly assigned to the saline group (control group) (n = 12) or the TS and comorbid anxiety model group (model group) (n = 36).The model group was induced by injecting with IDPN (250 mg kg^−1^) once daily for seven consecutive days (18). The saline group was injected with an equal volume of 0.9% saline (15 ml kg^−1^) by intraperitoneal injection. The rats in the model group were provided water at regular times (9:00 am to 9:10 am, and 9:00 pm to 9:10 pm) simultaneously during the 7 days. The rats in the model group were given an empty water bottle stimulation randomly each day during the watering periods to induce the emotional stress for 14 consecutive days, while rats in the control group were allowed to get purified water freely (20). Twenty-one days later, The TS and comorbid anxiety model group was further divided into three groups by evaluating grades of stereotypy (21): the model group (n = 12), the fluoxetine hydrochloride (FLX) group (n = 12), and the JPZDD group (n = 12). Then, all the rats were fed *via* gavage for four consecutive weeks. Series of behavioral tests were conducted by stereotypy test, open field test, and elevated plus maze. At last, the rats were euthanized.

### Drugs and Reagents

JPZDD granules were provided by the Pharmacy Department of the Third Affiliated Hospital of Beijing University of Chinese Medicine identified by Shu Lu. Ten different Chinese medical herbs were included in JPZDD (**Table 1**). A pair of JPZDD granule was dissolved in 50 ml of distilled water, after well-mixed, the solution was stored at 4°C before use. The control and model groups received gavage with 0.9% saline (10 ml kg^−1^), the FLX-treated group received gavage with FLX (4.2 mg kg^−1^ d^−1^) (0943A; Patheon France, Jiangsu, People's Republic of China), and the JPZDD group was fed *via* gavage with JPZDD (16 g kg^−1^ d^−1^) once daily during a period of 4 weeks.

**Table 1:** Contents of JPZDD.

Chinese name	Common name	Family	Weight(g)	Part used
Tai Zi Shen	Pseudostellaria heterophylla	Caryophyllaceae	10	root
Bai Zhu	Atractylodes macrocephala Koidz	Compositae	10	root
Fu Ling	Poria cocos Wolf	Polyporaceae	10	sclerotium
Ban Xia	Pinellia ternata Breit	Araceae	6	tuber
Chen Pi	Citrus reticulata Blanco	Rutaceae	6	peel
Fang Feng	Saposhnikovia divaricate Schischk	Umbelliferae	6	root
Long Dan Cao	Gentiana scabra Bge	Gentianaceae	3	root
Dang Gui	Angelica sinensis Diels	Umbelliferae	10	root
Chuan Xiong	Ligusticum chuanxiong Hort	Umbelliferae	6	root
Gou Teng	Uncaria rhynchopylla Jacks	Rubiaceae	10	stem

### Behavioral Studies

#### Stereotypy Recording

Stereotypy assessment was evaluated by two trained observers as previously described (21). The observers were blinded to the group conditions, and they recorded the stereotypy data after observation of the rat's behavior. For evaluating the stereotypy, each rat was observed for 2 min after IDPN injection and drug administration at the end of 1, 2, and 4 weeks. We took each observation of the rat from each observer and calculated the mean as the average score and using that as the objective indicator of behavioral alterations.

#### Elevated Plus Maze

The elevated plus maze (EPM) test was conducted 1, 2, 4 weeks after oral administration. The rats were tested for EPM as described previously (20, 22). The paradigm consisted of a cross-shaped plastic apparatus, elevated 100 cm above the ground, with two opposite open arms (50 × 10 cm^2^) and two closed arms (50 × 10 cm^2^) surrounded by a black plastic wall, 15 cm tall. During the trial, the rat was placed in the middle of the maze and was allowed to freely explore the new environment for 3 min. The ratio of open arm entries and total arm entries was counted during the test.

#### Open Field Test

The rats' general locomotor and rearing activity were measured by an open field test (OFT) as described in Zhang et al. (20, 23). It is used to test the anxiety-related behavior. The apparatus was made of a wooden box (75 cm long × 75 cm wide × 40 cm high) with black walls. The floor of the box was divided into 25 equal squares with 1 cm wide black lines. Once the rat was placed at the centre, both locomotor activity (number of line crosses) and rearing activity (standing upright) were manually recorded over a 5-min period. Each rat was tested individually, and only once at the end of 1, 2, and 4 weeks.

### Ultrastructural Examination by Transmission Electron Microscopy

Sample preparation was carried out as previously described (24). First, the striatum and the prefrontal cortex tissues (n = 3) were cut into approximately 1-mm^3^ pieces and then the samples were fixed in 2.5% glutaraldehyde in 0.1 mol/L sodium phosphate buffer (pH 7.4) overnight at 4°C and osmicated in 1% osmium tetroxide for 2 h at 20°C. The sample was embedded in Epon812 and sectioned using a Leica UC7 ultramicrotome after dehydration using the published methods (24). The photographs of the sections were viewed with Transmission Electron Microscopy (TEM) using (Tecnai G 20 TWIN, FEI) at 200 kV.

### Enzyme-Linked Immunosorbent Assays

After the animals were euthanized, the blood was collected from the abdominal aorta and placed in Ethylene Diamine Tetraacetic Acid (EDTA)-coated tubes, kept on ice, and centrifuged at 3000 × *g* for 15 min at 4°C. The plasma was separated from the whole blood and spit into small tubes to store at −70°C. The GABA and GLU levels in plasma were determined by enzyme-linked immunosorbent assays (ELISA) using a commercially available assay kit from Rui-Bo-Ge (Beijing, China). All procedures were performed strictly according to the instructions provided in the kit.

### High-Performance Liquid Chromatography

High-performance liquid chromatography (HPLC) was performed using ESA 5600A HPLC with model 5600A CoulArray Detector-8, and ESA MD-15 column (3.2 × 150 mm, 5 µm). Data were collected and analyzed with ESA Software Version. Separation of analysis was performed using a Waters XterraTM MS column (3.0 × 50 mm, 2.5 µm, Part 186000598), preceded by a pre-column filter (Shiseido, Guard Cartridge, Capcell C18 MG S-5, 4.0 × 10 mm). The mobile phase consisted of methanol (20%), acetonitrile (3.5%), disodium hydrogen phosphate (100 mM) (pH 6.7, adjusted by phosphoric acid). Woking solutions of GABA (G1251; Sigma, St. Louis, MO, USA) and GLU (5835, sigma, St. Louis, MO, USA) (40, 20, 10, 5, 2.5, and 1.25 μg/ml) were used. The column temperature was maintained at an ambient temperature of 40°C. The flow rate was set to 0.6 ml min^−1^.

### Protein Extraction and Western Blot for GABAAR and NMDAR

The left striatum and cortex of rats (n = 3, per group) were homogenized in an ice-cold radio immunoprecipitation assay buffer (RIPA) (C1053; Applygen, Beijing, People's Republic of China) and protease inhibitor (4693124001; Hoffman-La Roche Ltd., Basel, Switzerland), followed by being centrifuged at 10,000*g* for 10 min at 4°C.Then the supernatants was collected. Bicinchoninic acid (P1511; Applygen) was used to quantify the protein concentrations in the supernatants. Proteins (40 μg) were separated with 10% SDS-polyacrylamide gel electrophoresis and transferred to polyvinylidene fluoride (PVDF) membranes. After washing with Tris-buffered saline, the PVDF membranes were blocked with 5% skimmed milk powder for 1 h, and incubated overnight at 4°C with the appropriate primary antibody (ab193311 and ab52177; Abcam, Cambridge, UK) at 1:1,000 and 1:3,000 dilution recommended by the supplier. The membrane was then washed three times in Tris-buffered saline with Tween 20, and primary antibodies were detected with secondary antibodies (goat anti mouse IgG, ZB-2305; goat anti rabbit IgG, ZB-2301; ZSGB-BIO, Beijing, People's Republic of China) at a dilution of 1:1,000 and 1:2,000 conjugated to horseradish peroxidase for 1 h at room temperature. The protein bands were visualized and analyzed with Super ECL Plus Detection Reagent (sc-2048, Santa Cruz Biotechnology Inc., Dallas, TX, USA) and Quantity One software (Bio-Rad Laboratories Inc., Hercules, CA, USA).

### Real-Time Polymerase Chain Reaction for GABAAR and NMDAR

The total ribonucleic acid (RNA) from the striatum and cortex (n = 6, per group) were isolated with Trizol reagent according to the manufacturer protocol (15596018; Thermo Fisher Scientific, Waltham, MA, USA). GABAAR α2 gene primers were as follows: forward primer, 5′- TGGCTGAACAGAGAATCGGT-3′ and reverse primer, 5′- GGGAAGGGAATTTCGAGCAC-3′. NMDAR1 primers were as follows: forward primer, 5′- TCCGTGGACATCTACTTCCG-3′, and reverse primer, 5′- AGATAAAGGCGTGCAGCTTG-3′. GAPDH primers were as follows: forward primer, 5′- CAACTCCCTCAAGATTGTCAGCAA-3′ and reverse primer, 5′- GGCATGGACTGTGGTCATGA-3′. The basic protocol for real-time polymerase chain reaction (RT-PCR) was carried out as previous described (18). After initial denaturation at 94°C for 10 min, 45 cycles of amplification followed. The cycles were conducted at 94°C for 15 s and at 60°C for 60 s in order to cDNA amplification. At last, the final elongation was at 72°C for 10 min. Then a RT-PCR machine (ABI7500; Thermo Fisher Scientific) was used to detect the SYBR green signal. The PCR products were analyzed by gel electrophoresis and melting curve analysis to confirm specific amplifications. Messenger ribonucleic acids (mRNA) expressions were normalized to GAPDH. Transcript levels were quantified using the 2-ΔΔCt-value method.

### Statistical Analysis

Data shown in the article were expressed as the mean ± standard deviation and analyses performed using IBM SPSS 20.0 statistical software (SPSS Inc., Chicago, IL, USA). Statistical analysis was performed using either one-way analysis of variance (ANOVA) (equal variance) or a Welch's ANOVA (unequal variance) test. Differences were considered to be statistically significant for *p*< 0.05.

## Results

### Effects of JPZDD on TS and Comorbid Anxiety Models

#### Stereotypy Recording

**Figure 1:**
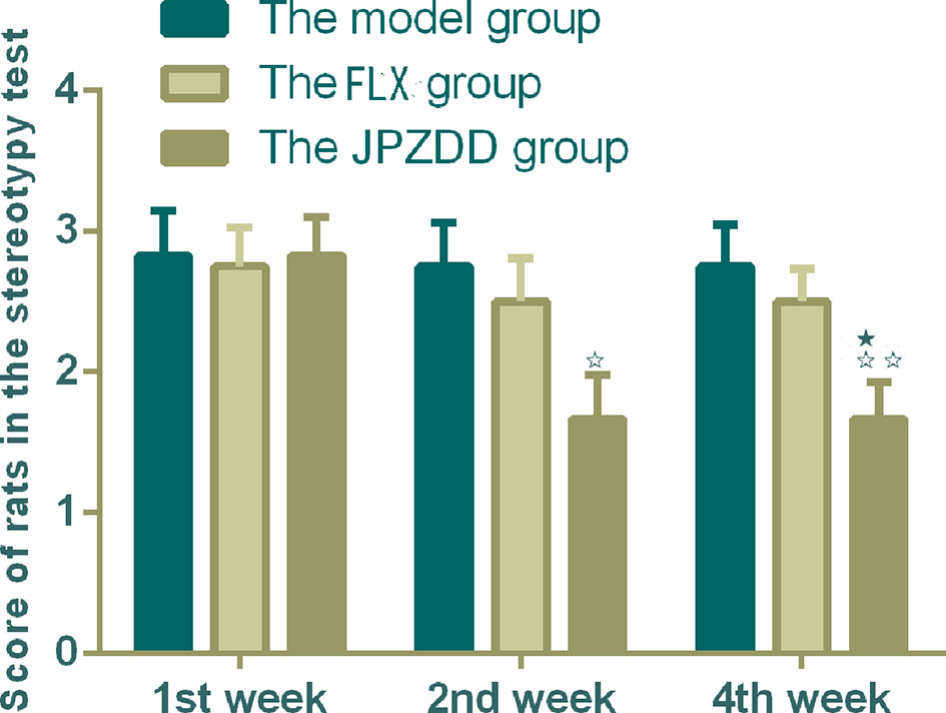
Effect of treatments on the stereotypy behavior score of TS and comorbid anxiety rats. Data are presented as mean ± standard deviation. (n = 12; 1 week: F = 0.03, P = 0.97; 2 weeks: F = 3.36, P = 0.05; 4 weeks: F = 4.56, P = 0.02); ^☆☆^P < 0.01, ^☆^P < 0.05 vs. the model group; ^★^P < 0.05 vs. the FLX group; Model group, TS and comorbid anxiety model group; FLX group, fluoxetine hydrochloride group; JPZDD group, Jian-Pi-Zhi-Dong Decoction group.

According to the criterior of stereotypy observation, the score of rats in the control group were zero. They took on normal activity (21). Compared with the control group, rats in the model group showed abnormal stereotypy behaviors before the treatment (*P* < 0.05). There was no difference between the model group and the treatment groups at week 1. Two weeks after treatment, the stereotypy scores for the JPZDD group showed a significant decrease compared with the model group from week 2 to week 4. (*P* < 0.05, *P* < 0.01). The Stereotypy score for the JPZDD group decreased significantly compared with the FLX group (*P* < 0.05) at week 4 (**Figure 1**).

#### Elevated Plus Maze Test

**Figure 2:**
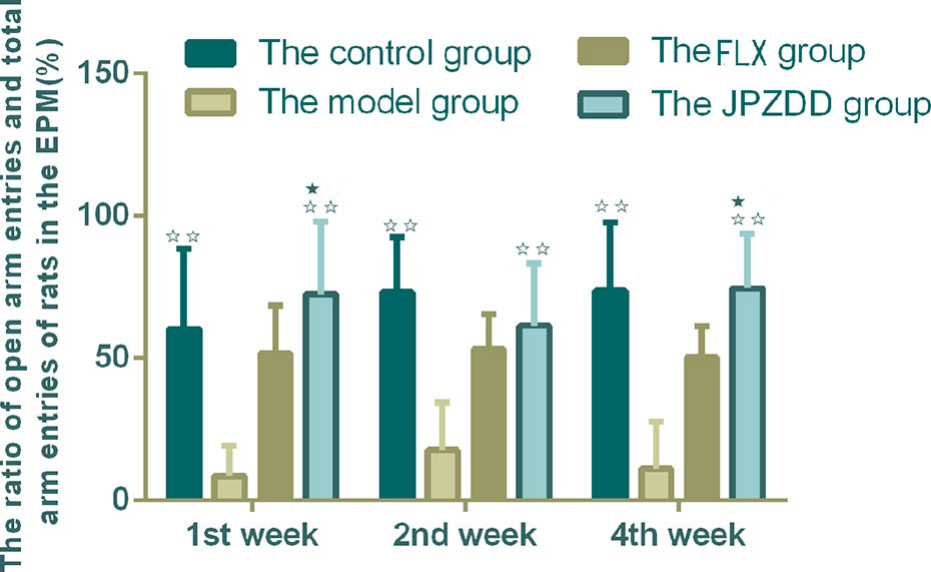
Effect of treatments on the elevated plus maze score of TS and comorbid anxiety rats. Data are presented as mean ± standard deviation. (n = 12; 1 week: F = 6.32, P = 0.00; 2 weeks: F = 7.2, P = 0.00; 4 weeks: F = 7.28, P = 0.00) ^☆☆^ P < 0.01 vs. the model group; ^★^ P < 0.05 vs. the FLX group; Model group, TS and comorbid anxiety model group; FLX group, fluoxetine hydrochloride group; JPZDD group, Jian-Pi-Zhi-Dong Decoction group.

The ratio of open arm entries and total arm entries (OE/TE) in the model group decreased significantly compared with the control group from week 1 to week 4 (*P* < 0.01). After the treatment, the JPZDD group showed increased OE/TE compared with the model group at each of the three points (*P* < 0.01). At week 1 and week 4 time points, the ratio of OE/TE increased significantly in the JPZDD group compared with the FLX group (*P* < 0.05) (**Figure 2**).

#### Open Field Test

**Figure 3:**
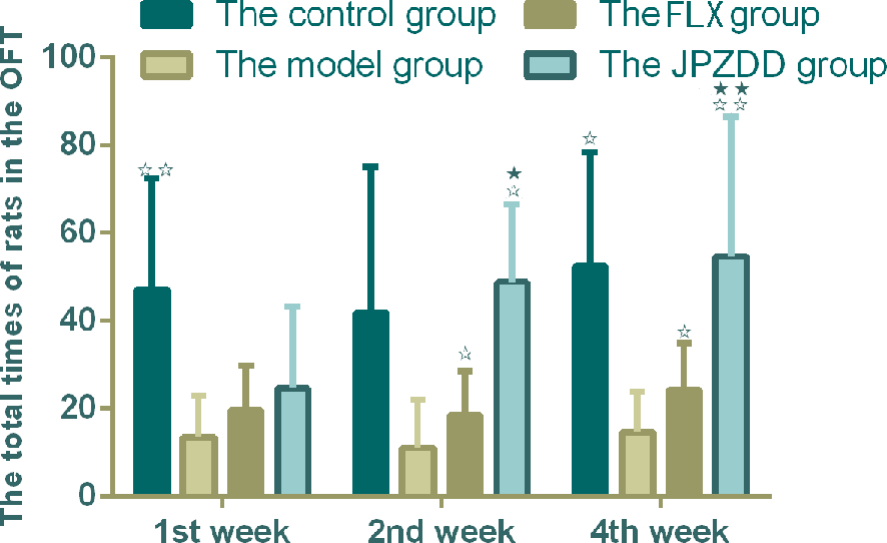
Effect of treatments on the OFT of TS and comorbid anxiety rats. Data are presented as mean ± standard deviation. (n = 12; 1 week: F = 18.06, P = 0.00; 2 weeks: F = 2.08, P = 0.04; 4 weeks: F = 19.38, P = 0.00) ^☆☆^ P < 0.01, ^☆^P < 0.05 vs. the model group; ^★★^ P < 0.01, ^★^ P < 0.05 vs. the FLX group; Model group, TS and comorbid anxiety model group; FLX group, fluoxetine hydrochloride group; JPZDD group, Jian-Pi-Zhi-Dong Decoction group.

The OFT showed that the locomotion/exploratory behavior of the rats in the model group decreased significantly compared with the control group at week 1 and week 4 (*P* < 0.01, *P* < 0.05). The locomotion behavior in both the JPZDD group and the FLX group increased after the treatment, and there was a statistical difference between them at week 2 and week 4 (both *P* < 0.05) (**Figure 3**).

### Effects of JPZDD on Mitochondrial Ultrastructure Changes in the Cortex and Striatum of the Brain

**Figure 4:**
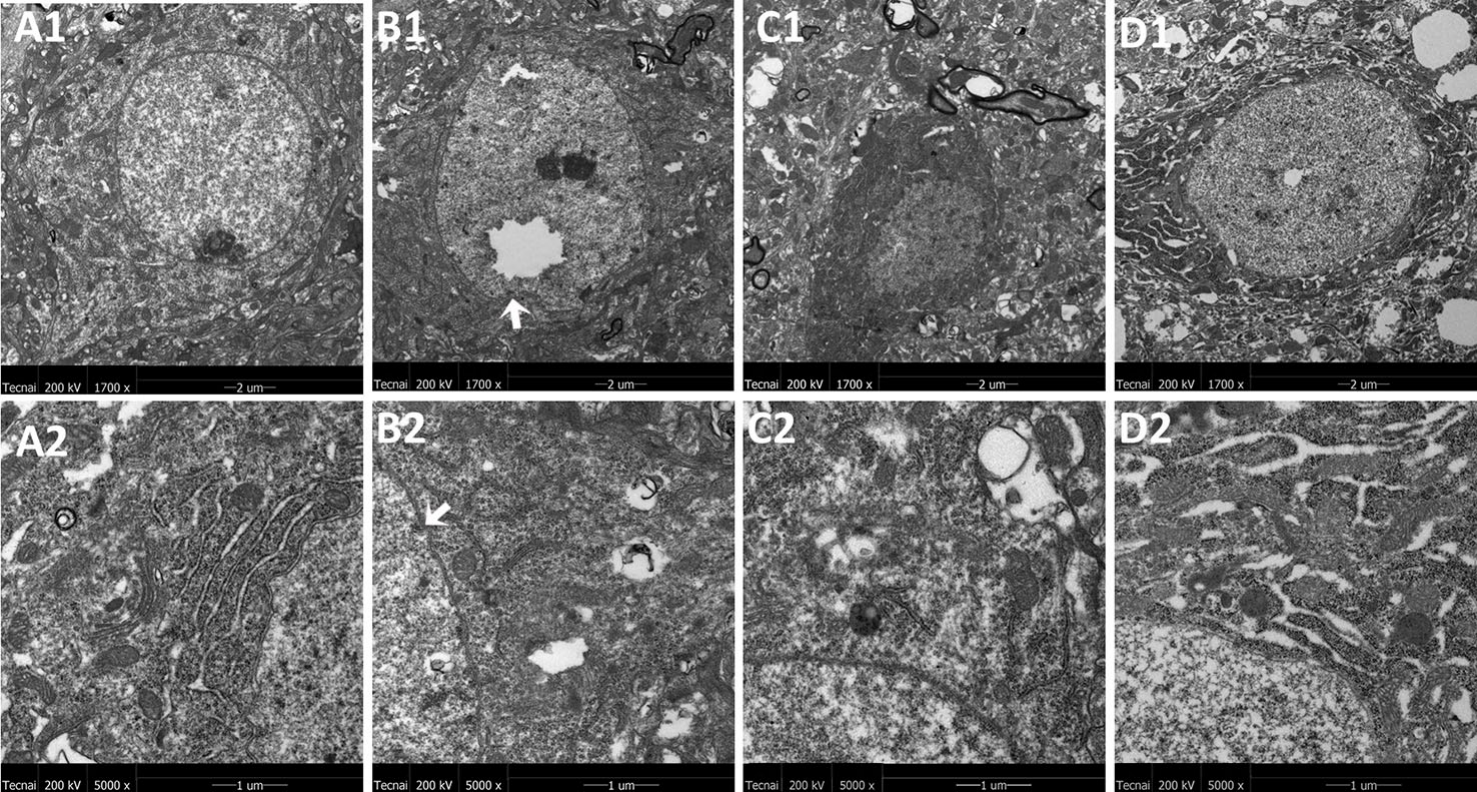
Mitochondria abnormalities identified in the frontal cortex region by transmission electron microscope. n = 3 for each group. Representative image of each group was viewed at 1,700 × (scale bar = 2 μm) and 5,000 × (scale bar = 1 μm) magnifications to detect changes in the frontal cortex mitochondria. A1: Representative image of low magnification (1,700×) image from control group. B1: Representative image of low magnification (1,700×) image from model group; The white arrow indicates mitochondria abnormalities. C1: Representative image of low magnification (1,700×) image from FLX group; D1: Representative image of low magnification (1,700×) image from JPZDD group; A2: Representative image of high magnification (5,000×) image from control group. B2: Representative image of high magnification (5,000×) image from model group; Abnormal mitochondrion is shown by the white arrow. C2: Representative image of high magnification (5,000×) image from FLX group; D2: Representative image of high magnification (5,000×) image from JPZDD group.

The representative ultrastructural micrographs of the prefrontal cortex for each group are shown in **Figure 4**. The organelles were rich. The membrane of the mitochondria in the control group seemed smoothly and clearly; the mitochondrial cristae could be seen clearly and was properly ordered (A1, A2). By contrast, in the model group, the boundary of the nuclear membrane was unclear, the cytoplasm was swollen, and mitochondrial dysfunction could be seen (such as swollen cristae), there were numerous irregular mitochondria, and part of the mitochondrial membrane was absent. Also, the axonal alignment was disordered and swelling could be seen (B1, B2). All treatments reversed these alterations (C1, C2, D1, D2).

**Figure 5:**
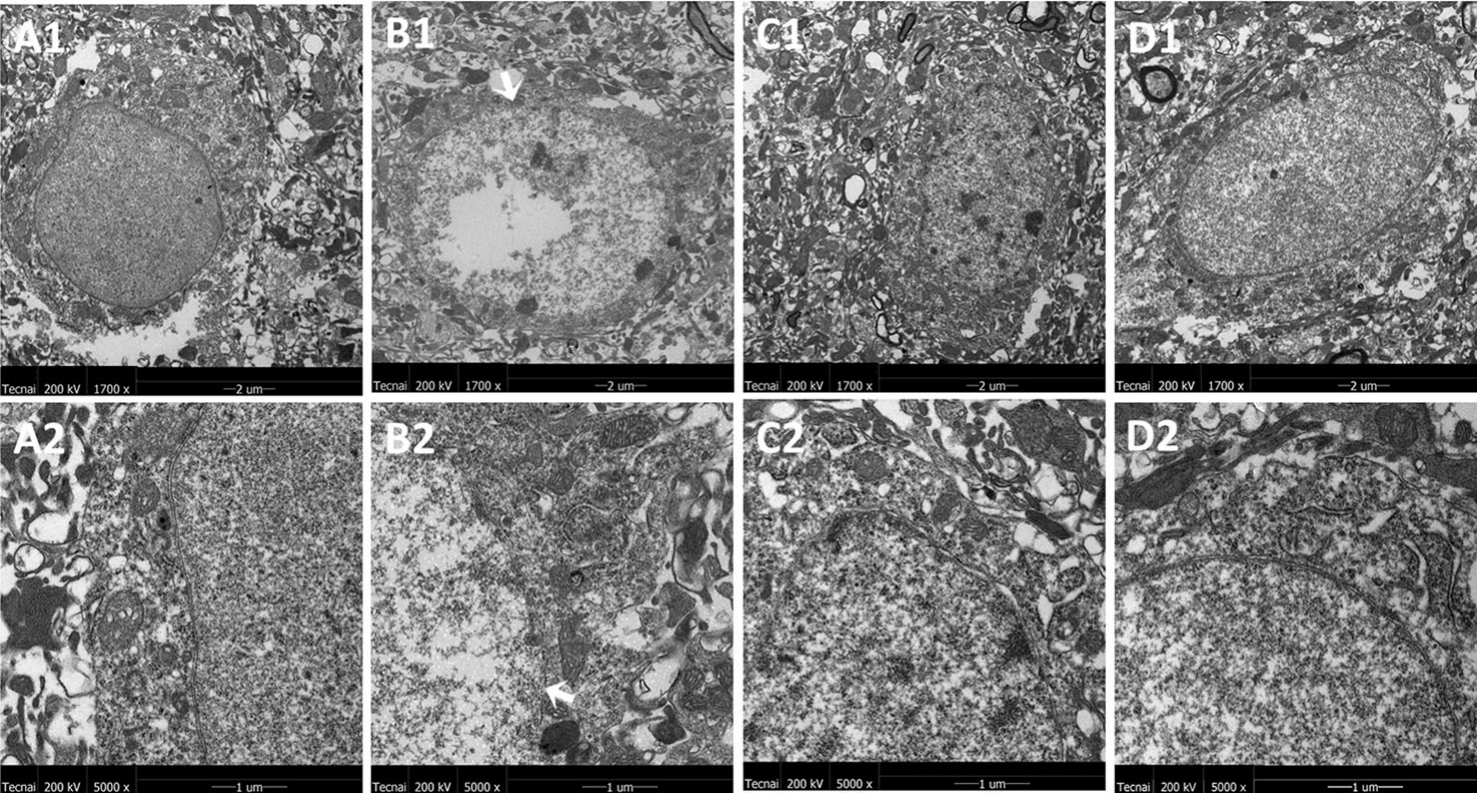
Mitochondria abnormalities identified in the striatum region by transmission electron microscope. n = 3 for each group. Representative image of each group was viewed at 1,700× (scale bar = 2 μm) and 5,000× (scale bar = 1 μm) magnifications to detect changes in the striatum mitochondria. Abbreviations: A1: Representative image of low magnification (1,700×) image from control group. B1: Representative image of low magnification (1,700×) image from model group; The white arrow indicates the rupture of the double membrane. C1: Representative image of low magnification (1,700×) image from FLX group; D1: Representative image of low magnification (1,700×) image from JPZDD group. A2: Representative image of high magnification (5,000×) image from control group. B2: Representative image of high magnification (5,000×) image from model group; “Disrupted” membrane is shown by the white arrow. C2: Representative image of high magnification (5,000×) image from FLX group; D2: Representative image of high magnification (5,000×) image from JPZDD group.

The representative ultrastructural micrographs of striatum for each group are shown in **Figure 5**. In the control group, the neuron was rich, the structure normal, and the mitochondrial cristae was clearly visible and properly ordered (A1, A2). In the model group, significantly higher numbers of mitochondrial abnormalities were consistently observed, and the rupture of the double membrane and the destruction of the internal cristae could be seen (B1, B2). The FLX group (C1, C2) and the JPZDD group (D1, D2) experienced a reverse in these alternations after treatment.

### Effects of JPZDD on the Expression of Amino Acid Neurotransmitters in Serum

**Figure 6:**
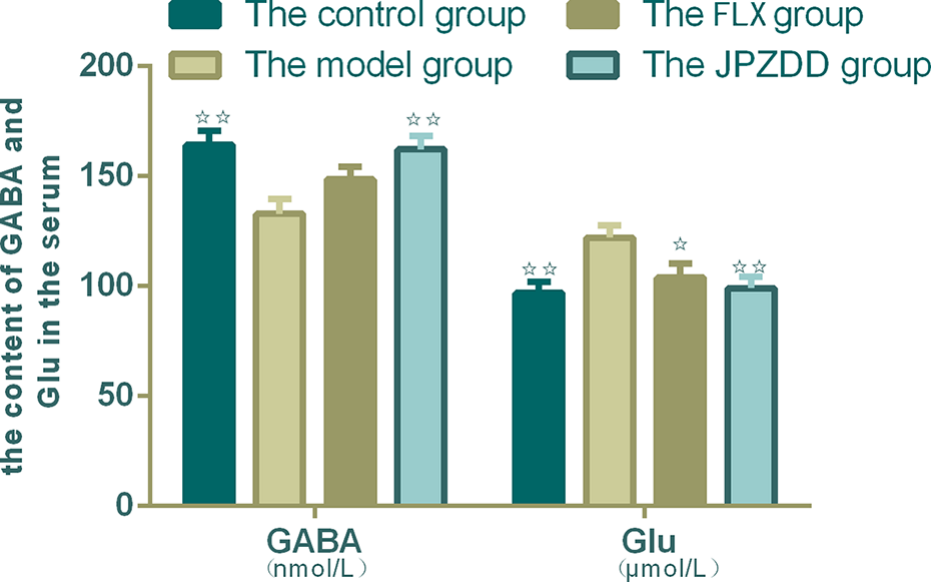
Effect of JPZDD on expression of GABA and GLU in the serum. Data are presented as mean ± standard deviation. n = 10 for the control group, FLX group, and JPZDD group; n = 12 for the model group (GABA F = 5.785, P < 0.01; GLU F = 4.4.54, P < 0.01). ^☆☆^ P < 0.01, ^☆^ P < 0.05 vs. the model group; Model group, TS and comorbid anxiety model group; FLX group, fluoxetine hydrochloride group; JPZDD group, Jian-Pi-Zhi-Dong Decoction group.

Serum concentrations of GLU and GABA were confirmed by ELISA. The serum levels of GABA in the model group decreased than the control group (*p* < 0.01). The content of GABA in the JPZDD group increased significantly than in the model group (*p* < 0.01). GLU concentration in the model group was higher than the control group, and GLU levels in both the FLX group and JPZDD group decreased compared with the model group (*p* < 0.05, *p* < 0.01) (**Figure 6**).

### Effects of JPZDD on the Expression of Amino Acid Neurotransmitters in the Striatum

**Figure 7:**
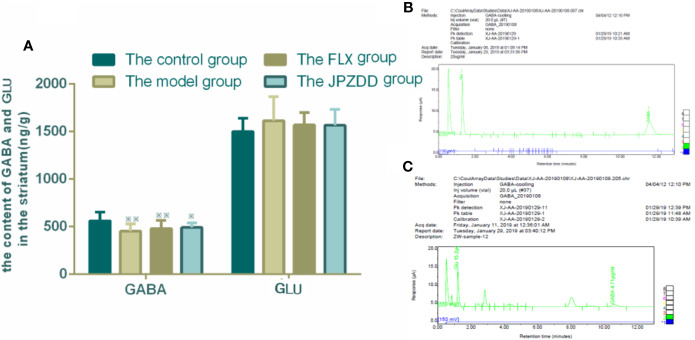
Effect of JPZDD on expression of GABA and GLU in the striatum. Data are presented as mean ± standard deviation. n = 12 for each group (GABA F = 4.19, P < 0.01; GLU F = 0.81, P > 0.05). ^※※^ P < 0.01,^※^ P< 0.05 vs. the control group; **(A)** The content of GABA and GLU in the striatum **(B)** Mixed amino acid standards **(C)** A sample of amino acids in brain striatum. Model group, TS and comorbid anxiety model group; FLX group, fluoxetine hydrochloride group; JPZDD group, Jian-Pi-Zhi-Dong Decoction group.

The concentrations of GLU and GABA of the striatum were also confirmed by HPLC. GLU and GABA were well separated with different peak value. Thus, we convinced that this method was specific credible for the measurement of two amino acids. GABA levels in the model group (451.89 ± 77.80) decreased compared with the control group (561.12 ± 92.75, p < 0.05). The GABA levels in the FLX and JPZDD groups showed an elevated trend compared with the model group, but there was no significant difference. And there was no statistical difference in GLU levels among the groups (**Figure 7**).

### Effects of JPZDD on the Expressions of GABAAR and NMDAR

**Figure 8:**
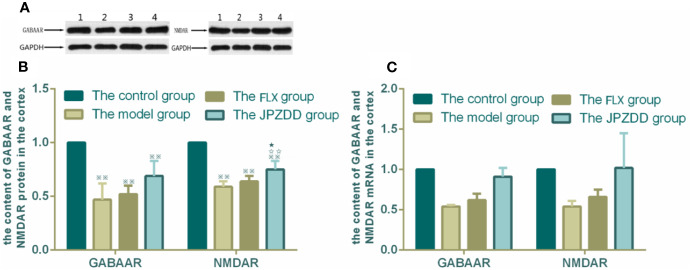
Effects of treatments on the expression levels of GABAAR and NMDAR in the cortex of TS and comorbid anxiety rats. WB and RT-PCR were performed to investigate changes in the expression of GABAAR and NMDAR and the effects of JPZDD on those levels in the cortex. ^※※^ *P*< 0.01 vs. the control group; ^☆☆^ *P* < 0.01 vs. the model group; ^★^ *P* < 0.05 vs. the FLX group. **(A)** representative immunoblots showing the effects of FLX and JPZDD on the expression of GABAAR and NMDAR in the cortex. 1, control group; 2, model group; 3, FLX group; 4, JPZDD group; **(B)** the content of GABAAR and NMDAR protein in the cortex. (n = 3 per group, *F* = 36.45, *P*=0.01; *F* = 34.6, *P* = 0.00). **(C)** GABAAR and NMDAR mRNA expression in the cortex (n = 6 per group, *F* = 0.348, *P* = 0.791; *F* = 0.499, *P* = 0.687). Model group, TS and comorbid anxiety model group; FLX group, fluoxetine hydrochloride-treated group; JPZDD group, Jian-Pi-Zhi-Dong Decoction group.

**Figure 9:**
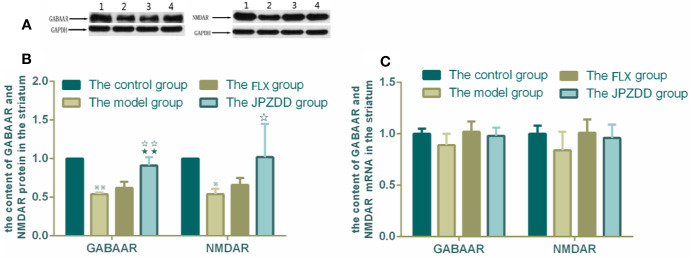
Effects of treatments on the expression levels of GABAAR and NMDAR in the striatum of TS and comorbid anxiety rats. WB and RT-PCR were performed to measure changes in the expression of GABAAR and NMDAR and the effects of JPZDD on those levels in the striatum. ^※※^ *P*< 0.01, ^※^ *P*< 0.05 vs. the control group; ^☆☆^ *P* < 0.01, ^☆^ *P* < 0.05 vs. the model group; ^★★^ *P* < 0.01, vs. the FLX group. **(A)** representative immunoblots showing the effects of FLX and JPZDD on the expression of GABAAR and NMDAR in the striatum. 1, control group; 2, model group; 3, FLX group; 4, JPZDD group; **(B)** the content of GABAAR and NMDAR protein in the striatum (n = 3 per group, *F* = 30.74, *P* = 0.00; *F* = 39.39, *P* = 0.00). **(C)** GABAAR and NMDAR mRNA expression (n = 6 per group, *F* = 2.62, *P* = 0.08; *F* = 2.07, *P* = 014). Model group, TS and comorbid anxiety model group; FLX group, fluoxetine hydrochloride-treated group; JPZDD group, Jian-Pi-Zhi-Dong Decoction group.

The protein expressions of GABAR and NMDAR in the cortex and striatum were confirmed by Western blot (WB) analysis. In the cortex, the expressions of GABAAR and NMDAR in the control group were 1.00 ± 0.00. The level of GABAAR and NMDAR in the model group were lower than the control group (0.47 ± 0.15 and 0.59 ± 0.05, both *P* < 0.01). The JPZDD group demonstrated the attenuated dissipation of GABAAR (0.69 ± 0.14, *P* < 0.05) and NMDAR (0.75 ± 0.08, *P* < 0.01). The difference between the JPZDD group and the FLX group was found in NMDAR test (**Figure 8**). In the striatum, the expression of GABAAR and NMDAR were lower in the model group (0.54 ± 0.02, 0.54 ± 0.07) than the control group (1.00 ± 0.00, 1.00 ± 0.00, *P* < 0.01). After treatment, the expression of GABAAR and NMDAR increased significantly in the JPZDD group (0.91 ± 0.11, 1.02 ± 0.43), compared with the model group (*P* < 0.01, *P* < 0.05). GABAAR expression in the JPZDD group was higher than the FLX group (0.62 ± 0.08, *P* < 0.01) (**Figure 9**).

GABAAR and NMDAR mRNA transcript expressions in the cortex and the striatum were measured by RT-PCR. Standard curves were drawn for the genes. No primer dimers or other PCR products were generated. There was no statistical difference among the groups in the cortex (**Figure 8**). The same trends can be seen in the striatum, but there was still no statistical difference among the groups (**Figure 9**).

## Discussion

Normally motor and phonic tics are the typical symptoms of TS. Apart from the tics, most patients with TS have associated neuropsychiatric comorbidities. Treatment of comorbid conditions is essential for all patients with TS, but until now, there has been a lack of studies on efficient therapeutic regimens. FLX is selected as the first-line drug to treat TS and comorbid anxiety disorders (25). In addition to being effective for the anxiety, there may be an indirect reduction in tic severity (26). But the adverse effect of FLX was more obvious in childhood, such as the symptoms of decreased food appetite, thirsty, insomnia, et al. Treatment with TCM can provide multiple therapeutic effects on multiple targets, which suggests its feasibility in the treatment of TS and comorbid anxiety.

According to traditional Chinese medicine, the heart houses the mind (shen) and the pathogenesis of TS and comorbid anxiety involves spleen Qi deficiency, liver wind stirring, and heart shen lost (27). Based on this theory, JPZDD was created to reinforce spleen Qi, smooth liver wind, and house heart shen. JPZDD is evolved by two ancient formulas of LJZT and XQW. The main function of LJZT is strengthening spleen Qi while XQW smoothing liver wind and housing heart shen. The compounds in the JPZDD may contribute to the effects of JPZDD in preventing and treating TS and comorbid anxiety. *Gentiana scabra* Bge could clear away the liver wind, and *Pseudostellaria heterophylla* could invigorate the spleen Qi, and they are the chief drugs. *Atractylodes macrocephala* Koidz, *Poria cocos* Wolf, *Pinellia ternata* Breit, and *Citrus reticulate* Blanco are adjuvant herbs. *Saposhnikovia divaricate* Schischk and *Uncaria rhynchopylla* Jacks, *Angelica sinensis* Diels, and *Ligusticum chuanxiong* Hort are assistant herbs. JPZDD took good effect in both anti-tics and improving the mood condition of patients with TS and comorbid anxiety in clinics. In previous studies, efforts were made to reveal the appropriate dose and the separated formula of JPZDD (28). We tried to find the target of JPZDD. We found that JPZDD played a crucial role in the abnormal behaviors of TS rats by keeping the balance of neurotransmitters in the CSTC circuit (18, 29), but the therapeutic mechanism underlying the activity of JPZDD in treating TS and comorbid anxiety is yet unknown. So in this study, we tried to investigate the function of the protective mechanism and the related mechanisms of JPZDD in model rats with TS and comorbid anxiety.

In our study, we established a TS and comorbid anxiety rat model by IDPN intraperitoneal injection combined with an uncertain empty bottle stimulation. Surface validity and structural validity were verified, and it could be used as an animal model for studying TS and comorbid anxiety (19). Behavioral tests, such as the stereotypy test, OFT, and EPM, were conducted. Stereotypy test is widely used in the evaluation of tic-like behavior, while EPM and OFT are reliable tests for investigation of anxiety-like behaviors in rodents. We found that the severity of stereotypy behavior in the JPZDD group alleviated significantly compared with the FLX group at week 4. This indicated that JPZDD positively correlated with a reduced number of tics. In the OFT test, the locomotion/exploratory behavior of the rats in the model group decreased significantly, while in the EPM test, the OE/TE also decreased in the model group. These indicated the rats in the model group showed anxiety. Our findings indicated that JPZDD significantly reversed the anxiety behavior of TS and comorbid anxiety rats in the OFT and EPM tests. From the results of those behavioral tests, we found JPZDD had a positive effect on both alleviating the degree of tic and anxiety. However, further studies should explore the multi-effect mechanism of JPZDD.

The exact pathophysiology between TS and comorbid anxiety is still unknown; however, they are thought to be partly related to dysfunction in the CSTC (2). The basal ganglia (caudate, striatum, and globus pallidus) play important role in the control of voluntary movements and abnormalities affecting these areas, and they can suffer from a variety of movement disorders, including TS (2). Different cortex areas can produce multiple effects, simple tics can be caused by the abnormal activation of the motor cortex, while complex tics can be associated with the abnormal activation of the premotor cortex. Structural changes in neurons of the striatum can be seen in neuropathological studies. Ablation of fast-spiking interneuron in the dorsal striatum in Tourette syndrome can produce anxiety (30). Several studies suggest psychiatric illnesses can be caused by mitochondrial dysfunction (31). Psychological stress can cause mitochondrial injuries in rats (32). The present study observed the ultrastructure changes of the rat's cortex and striatum. Subtle but significant changes in TEM demonstrated three things: the boundary of the nuclear membrane was unclear, the cytoplasm swelling, and mitochondrial dysfunction (such as irregular mitochondria and imperfect of the mitochondrial membrane) could be seen both in the cortex and striatum of the model group, and both FLX and JPZDD could reverse these alternations. The mitochondrial dysfunction not only impairs energy production, but also affects other key cellular processes. Impaired mitochondrial function might disrupt normal neural plasticity and reduce cellular resilience. And this could promote the development or progression of mood and psychiatric disorders (31). Although the cause of dysfunction in the basal ganglia structures is still obscure, enhancing mitochondrial function may represent a new approach for the development of treatment method for these complex disorders.

Moreover, abnormalities in CSTC circuit with an imbalance of excitatory and inhibitory neurotransmitters seem to have a significant effect on the pathogenesis of TS and its comorbidities (33). Neurotransmitters, including dopamine, GLU, serotonin, and acetylcholine, located within these pathways, are relevant in the pathogenesis of TS and comorbid disorders (8). The influences of dopamine and GLU are widely recognized in TS; however, inhibitory GABA loads which counterbalance to GLU transmission in the central nervous system may also play an influential role in the etiology of TS and comorbid anxiety. The interneurons of GABA are located in both the striatum and the cortex. Our research also pays more attention to the change of GABA in the striatum and prefrontal cortex in mood regulation. Abnormalities in the GABA pathway are postulated to cause the disinhibitive behavior in TS patients. High-anxiety rats had severe disturbances in response to stress owing to changes of neurotransmitters including GABA (34). GLU, the primary excitatory neurotransmitter, is the neurotransmitter of cortical and thalamic projection neurons and the subthalamic nucleus. Arguments supporting the role of the GLU system in TS include its essential role in CSTC pathways and extensive interaction between the glutamatergic and dopaminergic systems. A change of one neurotransmitter often influences the function of other interconnected transmitters significantly. The dynamic change between excitation and inhibition of the excitatory neuron mediates its excitatory neuronal plasticity, firing pattern and excitability, so the change of the neurotransmitters may be a dynamic indicator in the pathophysiology of tics.

Whether TS is associated with a hyper- or hypo-glutamatergic state is still controversial for lack of data support, therapeutic trials must consider both of these possibilities for this reason. Reduced levels of GLU have been identified in TS patients compared with controls. However, using 7T magnetic resonance spectroscopy, GLU level in children with TS was higher than the control group within the striatum and premotor cortex (9). The pathogenesis of TS is complicated including metabolic disturbances in excitatory and inhibitory neurotransmitters (35), and that this imbalance may result in changes of GLU and GABA serum levels. Both GLU and GABA are considered as biomarkers of TS (36). Is the change of amino-acid neurotransmitters in the serum consistent with the change of amino-acid neurotransmitters in the brain? So in this research, we examined the contents of GLU and GABA both in the serum and striatum and tried to reveal the truth. Our studies showed that an IDPN injection caused a significant decrease in GABA levels both in the striatum and serum of the model rats when compared with the control group. The GLU levels in the serum were higher in the model group than the control group. The neurotoxicity of GLU is mainly expressed in the following ways: damaging the cell membrane ion channels and causing cell edema, reducing mitochondrial functions, disturbing energy metabolism, and causing apoptosis in some cells (37). The alleviation of symptoms of tics and anxiety in TS and comorbid anxiety rats treated with JPZDD could be seen by the trend of increases of GABA levels in the striatum and the serum. The relative increase in GLU levels of the TS and comorbid anxiety exerted a neurotoxic effect, causing an excitatory state, while increased GABA may contribute to a tonic extra synaptic inhibition, interfering with the excitatory/inhibitory balance (18, 38) and improved the injury of the cerebral ultrastructure.

GABA is produced by GABAergic neurons and released at synapses, GABA mediate its brain excitability by the activation of its receptors. Benzodiazepines (which enhance the effects of GABA), are one kind of modulators of GABA A receptors, and are used widely in clinical studies (39). In the studies of TS patients, a reduced number of GABAergic interneurons in the striatum (14) and a decreased binding of GABA_A_ receptors in postmortem brains (40) indicated TS was associated with a reduced level of brain GABA by its receptors. In rodent and primate models, once the injection of GABA type A receptor antagonists disrupt the GABAergic connectivity, tic-like behaviors can be seen (38, 41). This work identified a decrease of GABAAR in the cortex and striatum of model rats, suggesting the GABA transmission was impaired. In addition, JPZDD exhibited stronger activity in the activation of GABAAR compared with the FLX group, especially in the striatum. A glutamatergic excitatory drive *via* the activation of *N*-methyl-d-aspartate receptor (NMDA) receptors (42). The presence of NMDA receptors on dopaminergic neurons are thought to underlie memory and habit learning (43). Or rather, damaging NMDA receptors did not prevent goal-directed but habitual learning (44). In a transmission disequilibrium study, NMDA receptors were associated with the pathogenesis of TS in Chinese Han trios (39). In this study, we also found that NMDAR was involved in the occurrence of TS and comorbid anxiety. The number of NMDAR available decreased in the model group both in the cortex and striatum. After treatment, the number of NMDAR in the JPZDD group increased significantly differently from the model group both in the cortex and striatum. The number of NAMDAR increased significantly in the cortex among the JPZDD group when compared to the FLX group. In this study, we proposed a hypothesis that the activation of neurotransmission at the NMDAR would be beneficial to alleviate TS symptoms and TS-associated conditions (12). Unfortunately, no change was found in the GLU levels in the striatum. But evidence provides support for the use of GLU modulators (45). Binding neurotransmitters to receptors is important for the activation of a downstream signaling pathway. Therefore, the reason for JPZDD treatment can alter the neurotransmitter levels mainly through the changes of the GABAAR or NMDAR in the striatum or cortex. However, the present study still has some limitations. First, these studies in animal models mainly focused on limited neurotransmitters and its one kind of receptor, other neurotransmitters and receptors remains to be known. Second, JPZDD is a compound prescription, the exact mechanism of each Chinese herb in JPZDD should be validated by fingerprint of traditional Chinese medicine or pharmacokinetics in the future. Third, GLU and GABA are released by distinct neurons, so the structural and functional integrity of neurons is the basis of neurotransmitter release and regulation. Observing the ultrastructure of neurons by immunofluorescence would have important significance. We will continue our research in the future.

In brief, our present results provided new data for the preventive mechanism of JPZDD on TS and comorbid anxiety by molecular, biological methods. We also observed the mitochondrial changes in the rat brain. We found JPZDD exerted favorable effects on the rat model with TS and comorbid anxiety. We concluded that the beneficial effect of JPZDD was associated with keeping the balance of GLU and GABA neurotransmission by regulating its receptor expression and reversing mitochondrial ultrastructure changes. However, the exact function of mitochondria and the contributions of other signaling pathways and inhibitors related to the neuroprotective effects of JPZDD are the subject of ongoing studies.

## Conclusions

Comorbidity, or the presence of more than one disorder in an individual, is a prevalent condition affecting of the global population with TS. The purpose of our study was to determine if traditional Chinese medicine, specifically the compound JPZDD, could ameliorate or lower the presence of physical and phonic tics and comorbid anxiety. The findings of this study suggest that JPZDD can alleviate not only the stereotypy behavior but also anxiety behavior in rats with TS and comorbid anxiety. The related mechanisms might be associated with increases in the GABA level and decreases in the GLU level in the serum, as well as an increase in the striatal GABA level by activating GABAAR. JPZDD treatment also reverses mitochondrial dysfunction, both in the striatum and cortex in affected animals. In summary, the finding of this study provides new ideas into the pharmacological potential for using JPZDD in the treatment of TS and comorbid anxiety.

## Data Availability Statement

The raw data supporting the conclusions of this article will be made available by the authors, without undue reservation, to any qualified researcher.

## Ethics Statement

All the experimental animal procedures conformed to the guidelines of the Beijing University of Chinese Medicine Animal Care and Use Committee. Experimental protocols were approved by the Animal Experimentation Ethics Committee at the Beijing University of Chinese Medicine.

## Author Contributions

WZ contributed to the interpretation of results and writing of manuscript. WY contributed to the interpretation of results and data analysis. XL participated in the study design. QW and XB conducted the experiments. XC and SW reviewed and approved the manuscript.

## Funding

This work was supported by the Beijing Municipal Natural Science Foundation (7182101, XC), the National Natural Science Foundation for Young Scholars of China (no 81503615, WZ) and Cultivation plan of young famous doctors of Beijing University of Chinese Medicine (2019, WZ).

## Conflict of Interest

The authors declare that the research was conducted in the absence of any commercial or financial relationships that could be construed as a potential conflict of interest.

## Abbreviations

TS, Tourette syndrome; ADHD, attention deficit hyperactivity disorder; JPZDD, Jian-Pi-Zhi-Dong decoction; FLX, fluoxetine hydrochloride; GLU, Glutamate; GABA, γ-aminobutyric acid; GABAAR, GABA receptors Type A; CSTC, cortico-striato-thalamo-cortical; OFT, open field test; EPM, elevated plus maze; IDPN, 3,3-iminodipropionitrile; ELISA, enzyme-linked immunosorbent assay; PVDF, polyvinylidene fluoride; RNA, ribonucleic acid; mRNA, messenger ribonucleic acid.
